# Pilot Testing a Narrative Psychoeducational Video to Reduce Stigma and Improve Mental Health Literacy and Help-Seeking Among Latinx Adults: Examining Gender Differences and Contextual Insights

**DOI:** 10.1007/s10597-025-01584-4

**Published:** 2026-02-27

**Authors:** Daniel Hernan Saravia, Yesenia Aguilar Silvan, Jonathan I. Martinez

**Affiliations:** 1 Department of Psychology, University of California, Los Angeles, Los Angeles, United States; 2 Department of Psychology, California State University, Northridge, Northridge, United States

**Keywords:** Latinx mental health, Stigma, Mental health literacy, Help-Seeking intentions, Service use barriers

## Abstract

Despite similar rates of mental illness, non-Latinx Whites (NLW) received care at higher rates compared to Latinxs (National Institute of Mental Health, 2024). Latinx adults report lower mental health literacy (MHL) and higher stigma compared to NLW, which can explain treatment disparities, as both are linked to lower service utilization by Benuto et al. (Community Mental Health Journal, 56(7):1275–1283, 2019) and Mendoza et al. (International Journal for the Advancement of Counseling, 37(3):207–222, 2015). Given how these factors influence service use, our study examined the effect of a culturally informed psychoeducational video intervention on increasing MHL and reducing stigma among a non-college-educated Latinx adult sample, and whether this relationship depended on gender. We also assessed whether these changes in MHL and stigma led to increased help-seeking intentions. Results revealed that the video successfully increased MHL among the full sample and, unexpectedly, increased perceived stigma among Latinx females. Changes in MHL and stigma did not result in increased help-seeking intentions. To better understand these findings, we conducted a qualitative analysis of two open-ended questions, which provided context for why the video was effective in increasing MHL and why it unexpectedly increased perceived stigma in females. Additionally, the qualitative results offered insights into other factors that might influence Latinx adults’ help-seeking intentions. These findings will guide the development of future psychoeducational videos tailored for a Latinx audience, potentially helping to improve access to and utilization of mental health services.

Recent estimates show 18.9% of U.S. adults (46.6 million) have a mental illness, but less than half receive needed mental health services (NIMH, 2024). The unmet need is greater among ethnic minorities, especially Latinx adults, with only 32.6% receiving formal MH services compared to 48% of Non-Latinx Whites (NLWs), despite similar prevalence rates (Benuto et al., 2019; Cook et al., 2017; NIMH, 2024; Olfson et al., 2023). From 2019 to 2021, service utilization increased by 10% for NLWs but only 1% for Latinx, highlighting the treatment gap (Terlizzi & Schiller, 2022).

Research over recent decades, aligned with help-seeking models (Ajzen, 1991; Cauce et al., 2002), emphasizes mental health literacy (MHL) and stigma as key factors influencing Latinxs’ decisions to seek services (Forcén et al., 2023; Green et al., 2021). MHL refers to individuals’ broad understanding of mental health care and disorders (Jorm et al., 1997). Overall, MHL is strongly linked to service utilization, with longitudinal data showing that higher MHL is associated with a greater likelihood of using mental health services (Bonabi et al., 2016). Among Latinx adults, MHL is lower compared to NLW, which potentially explains the lower service utilization in this group. Latinxs MHL limitations often presents in the misattributing psychological disorders, recognizing MH symptoms, and knowing where and how to access MH services (Bignall et al., 2015; Jang et al., 2011; Uebelacker et al., 2012). Limited MHL among Latinx can be potentially understood by considering the limited and often absent conversations about this topic that may prevent greater familiarity with mental health challenges and available resources. For example, evidence from a qualitative study shows that Latinx young adults (18–25) reported a lack of exposure to MH information. Additionally, participants noted that MH is often ignored and treated as something that does not exist (Crowe et al., 2022). Findings from this study highlight the limited conversations about MH broadly, which helps contextualize the low levels of MHL that exist among Latinxs as well as the need to further provide content to increase Latinx MHL.

Stigma, defined as a negative belief linked to a personal characteristic and/or topic (i.e., MH), which can lead to discriminatory behavior (American Psychological Association, 2018), is associated with lower help-seeking behavior among Latinxs (Cabassa & Zayas, 2007; Interian et al., 2007). Stigma occurs at both the community level (perceived) and the individual level (personal), both of which significantly affect help-seeking behavior. Broadly, perceived stigma refers to an individual’s beliefs about societal attitudes toward MH (Fox et al., 2018), while personal stigma involves internalizing these beliefs and adopting societal attitudes about MH as one’s own (Vogel et al., 2013). Both have been found to dissuade help-seeking behaviors and service utilization among Latinx individuals. Specifically, perceived stigma decreases the likelihood of Latinxs seeking services, a pattern observed among Latinxs who recognize a need for treatment (Chavira et al., 2017; Dixon De Silva et al., 2020; Rastogia et al., 2012; Young & Rabiner, 2015). Similarly, higher personal stigma appears to hinder help-seeking and the ability to recognize the need for treatment (Fripp & Carlson, 2017; Mendoza et al., 2015). Latinxs consistently report higher levels of stigma compared to NLWs (Benuto et al., 2019; Cheng et al., 2018; Wong et al., 2019). The consideration of stigma is a priority among this Latinx population, as previous studies have found that stigma is often the most cited barrier to care (Menendez et al., 2019), and MH is often viewed as a sign of weakness or being crazy (Crowe et al., 2022). Given the influence of MHL and stigma on service use, mitigating these factors can promote more equitable MH service utilization.

Psychoeducational interventions effectively improve MHL and reduce stigma (Cabassa et al., 2012; Dueweke & Bridge, 2017; López et al., 2009). Although a proven resource, a recent systematic review found that only seven studies have implemented psychoeducational interventions specifically designed for Latinx adults since 2000, highlighting the dearth of culturally relevant options for Latinxs (Pérez-Flores & Cabassa, 2021). Of these interventions, four focus on addressing MHL and stigma related to depression. Given the harmful effects of depression on Latinx adults (Rosas et al., 2025), it is essential to continue developing and testing psychoeducational interventions that effectively target depression-related MHL and stigma.

Existing psychoeducational interventions provided to Latinxs have primarily depended on distributing printed materials, such as fotonovelas and brochures, in classroom settings (Cabassa et al., 2012; Unger et al., 2013) or through group sessions (Hernandez & Organista, 2013; López et al., 2009). However, recent data suggests that shifting from the traditional brick-and-mortar approach to digital video-based formats may be more effective. Specifically, in their scoping review on digital video interventions and mental health literacy, Ito-Jaeger et al. (2022) found that digital videos can improve MHL, reduce perceived and personal stigma, increase help-seeking intentions, and add value in terms of social validity. Additionally, video-based psychoeducational interventions have been shown to be more engaging and easier to disseminate (Ramos & Chavira, 2022), making them a promising tool for advancing MHL. Additionally, these videos may strongly resonate with young adults, who frequently search the internet and social media for health-related information (Lim et al., 2022).

Video-based psychoeducational interventions specifically tailored for Latinx communities are scarce (Gonzalez & Benuto, 2022; Lopez et al., 2009; Pérez-Flores & Cabassa, 2021). The current video-based psychoeducational interventions for Latinxs are usually under 10 min long, utilizing formats such as educational (e.g., slide-based presentations), interview (e.g., personal testimonials), and narrative storytelling (e.g., fictional accounts of mental health struggles) (Gonzalez & Benuto, 2022; Lopez et al., 2009). Incorporating narrative storytelling elements common in traditional printed materials, like fotonovelas, is essential when designing video interventions for Latinx groups. Narrative-driven methods, often developed with input from the target audience, have proven effective in increasing MHL, including better symptom recognition and awareness of available treatments, while also reducing stigma surrounding mental health disorders and the use of mental health services (Gonzalez & Benuto, 2022; Sanchez et al., 2019).

To our knowledge, only one video-based psychoeducational intervention has been specifically developed for Latinx audiences with internalizing disorders. In their study, Gonzalez and Benuto (2022) found that a narrative-based, video psychoeducational intervention informed by Latinas increased depression literacy and decreased depression stigma—serving as proxies for MHL and stigma, respectively. While these findings provide evidence for the effectiveness of video-based interventions, this evidence has been observed only among Latinas, leaving questions about their effectiveness for Latino men and limiting generalizability. This is a notable gap, as Latino men report lower rates of MH service use compared to Latinas and experience higher levels of stigma (Cho et al., 2014; Eisenberg et al., 2011; Pattyn et al., 2015). These disparities underscore the need to design video-based psychoeducational interventions that are inclusive of Latinos and the wider Latinx community. Existing video interventions for depression primarily promote help-seeking by discussing symptoms and treatment, but they often overlook the developmental course and early onset of MH disorders, which typically start in childhood. Educating viewers on the development, onset, and early warning signs of these conditions can facilitate earlier help-seeking and intervention, potentially preventing the progression to more severe or chronic MH issues (Mhango et al., 2023; Miklowitz, 2023). Current interventions often overlook co-occurring substance use disorders, which are estimated at 40.3% among those with depression. This underscores the need for psychoeducational content addressing these comorbid conditions (Pettinati et al., 2013).

## Current Study

Our study evaluated a community-informed, culturally tailored, video-based psychoeducational intervention among non-college-educated Latinx adults—an underrepresented group, with only 20.4% holding a college degree (Office of Minority Health, 2024). Our first aim was to assess the intervention’s impact on MHL and stigma. We hypothesized that participants would demonstrate increased MHL and decreased stigma following the intervention. Given known gender differences in MHL and stigma, we also examined whether gender moderated changes in these outcomes over time. Additionally, we conducted qualitative analyses to explore whether similar gender differences emerged in the frequency of MHL and stigma-related codes. Our secondary aim was to determine whether changes in MHL and stigma were associated with changes in help-seeking intentions. We hypothesized that increased MHL and reduced stigma would be associated with greater help-seeking intentions. Qualitative data were also analyzed to better understand how gender, MHL, and stigma interact, and to identify additional factors influencing help-seeking among Latinx participants.

## Method

### Procedures and Participants

Data from Latinx adults was collected via Qualtrics’ research panel between November 2021 and February 2022. Registered Qualtrics panel members who identified as Latinx were contacted about the opportunity to partake in the study. Individuals completed a screening survey to determine if they met the inclusion criteria. Eligibility criteria included (a) identifying as Hispanic, Latinx, or of any other Spanish origin, and (2) not having ever attended a college or university. The study received ethical approval from the University Institutional Review Board.

Before participating, participants reviewed the study information and provided consent. After consenting, they completed a survey that included demographic questions and the MHL, stigma, and help-seeking intentions measures. After watching the psychoeducation video, they completed the same battery of MH surveys and responded to questions about their previous lifetime use of both professional (e.g., psychologist, psychiatrist) and alternative (self-help groups, spiritual advisors, telephone hotlines, herbalists) MH service use. The study duration averaged 27 min. Upon completion, participants were awarded points through Qualtrics, which they could redeem for cash compensation, and received a debrief form of the study’s purpose.

### Stimulus

#### Narrative Storytelling Video: Victor Luna - My Journey Into Mental Healthcare

The research team developed an 8-minute narrative storytelling video influenced by feedback from community-based MH providers regarding how easily the video could be shared, how well it conveyed complex MH concepts, and the significance of storytelling for the Latinx community. The video drew on principles from a previously successful video that increased MHL, lowered stigma, and promoted help-seeking (Gonzalez & Benuto, 2022; Lopez et al., 2009; Sanchez et al., 2019). Specifically, the video used both a narrative and testimonial format—methods proven to effectively enhance MHL and reduce stigma—to tell the story of a fictional Latinx youth and his family as they struggle to understand the main character’s mental health issues. It also featured a testimonial where the lead character shares his journey into mental health care. Additionally, both formats included psychoeducational elements—such as illustrating common depression symptoms among youth and discussing therapy options and benefits. The video can be viewed at: https://youtu.be/z7nI1bqkp94?si=wCprEoIsHp0JUtCB.

### Measures

#### Demographic

The demographic questionnaire consisted of five items assessing participants’ age, marital status, employment status, income, and Latinx origin.

#### Depression Literacy Scale

The Depression Literacy Scale (D-Lit; Griffiths et al., 2004) is a 22-item questionnaire designed to assess various aspects of depression literacy. A sample item includes, *“Loss of confidence and poor self-esteem may be a symptom of depression.”* Responses are rated on a true/false scale. In this study, a subset of 10 of the original 22 items was administered. Some items were modified linguistically to improve clarity for participants. Additionally, items related to psychotropic medications were removed because this topic was not addressed. In our study sample, the average reliability (pre and post) of the scale was Kuder-Richardson (KR) = 0.523. The D-Lit measure served as a proxy for MHL and has similarly been used as a MHL proxy in previous study (Gonzalez & Benuto, 2022).

#### Depression Stigma Scale

The Depression Stigma Scale (DSS; Griffiths et al., 2004) is an 18-item questionnaire designed to assess both personal and perceived stigma related to depression. Items are rated on a 5-point Likert scale (1 = *Strongly Disagree* to 7 = *Strongly Agree*). A sample personal stigma item is, *“It is best to avoid people with depression so you don’t become depressed yourself.”* A sample perceived stigma item is, *“Most people believe that it is best to avoid people with depression so that you don’t become depressed yourself.”* In the present study, a subset of 13 of the original 18 items were administered. The item selection process mirrored the D-Lit Scale, where items covering topics not addressed in the study’s videos were removed. In our study sample, the average reliability (pre and post) of the scale was Cronbach’s α: 0.813.

#### Help-Seeking Intention

The MH Seeking Intention Scale (MHSIS; Hammer & Spiker, 2018) is a three-item questionnaire designed to assess an individual’s intention to seek help from a MH professional when experiencing a MH concern. Each item is rated on a 7-point Likert scale. The 3 items include, *“If I had a MH concern*,* I would intend/try/plan to seek help from a MH professional*,*”* rated from 1 (Extremely Unlikely) to 7 (Extremely Likely); In our study sample, the average reliability (pre and post) of the scale was Cronbach’s α: 0.907.

#### Perceived Challenges to Accessing Mental Health Care

To better contextualize our sample and gain insight into potential challenges faced when accessing MH care, two open-ended questions were asked: *“What do you believe is the biggest challenge when accessing MH care?”* and *“Do you believe speaking about MH is easy or difficult? Please explain.”* Participants provided a range of responses, from brief three-word answers to more detailed explanations consisting of several sentences.

## Data Analytic Plan

### Mixed Methods Design

A QUAN→qual mixed methods design was used (Palinkas et al., 2011) in which data collection began with a quantitative phase followed by a qualitative phase. The purpose of the quantitative analysis was to examine the effects of a video-based psychoeducational intervention on MHL, stigma, and help-seeking intentions, as well as explore gender differences. Findings from the quantitative analysis informed the subsequent qualitative analysis, which aimed to expand on the initial results by providing contextual insights about the observed relationships. Specifically, the qualitative analysis explored participants’ perspectives to deepen our understanding of how MHL and stigma influenced help-seeking, including gender differences, and to identify additional factors influencing help-seeking among Latinx participants that may not have been captured from the quantitative data alone.

### Quantitative Analysis

We conducted our analyses using IBM SPSS Statistics (Version 28; IBM Corp, 2021). We conducted two mixed factorial ANOVAs to evaluate the effectiveness of the narrative video intervention by examining (1) whether MHL increased and stigma decreased from pre- to post-intervention, and (2) whether these changes varied by gender. Time (pre and post) was a within-subject variable and gender (female and male) was a between-subject variable.

To assess whether changes in MHL and stigma from pre- to post-intervention predicted help-seeking intentions, we conducted a hierarchical regression analysis. In our first block, we entered our covariates variables, which included baseline help-seeking intentions, demographic variables such as sex, marital status, employment status, income variables commonly associated with help-seeking (Dunlap et al., 2016; Miller et al., 2016; Ojeda et al., 2006; Twomey et al., 2015).In our second block, we entered MHL and stigma change scores as our predictors. We computed change scores by subtracting post-intervention MHL and stigma scores from pre-intervention MHL and stigma scores.

### Qualitative Analysis

The coding team consisted of two Latine doctoral-level clinical psychology students (first and second authors). The coding team used an inductive (“bottom-up”) approach, creating codes based directly on the participants’ open-ended text responses without applying a pre-existing theoretical framework or apriori codes (Hsieh & Shannon, 2005). The coding team reviewed open-ended responses and identified initial patterns in the data. Preliminary codes were generated based on emerging concepts. These codes were categorized based on how responses were related and refined iteratively in weekly coding meetings. Definitions were developed for each code and category, which were then grouped into themes to create a final codebook. The codebook consisted of a label, definitions, and exemplar statements. The coding team double coded the open-ended responses based on the final codebook. Some responses addressed multiple topics and were therefore assigned up to two codes or categories.

Team-based coding consensus is an approach commonly used to address coding biases and ensure coding reliability (Cascio et al., 2019). Coding decisions were reviewed during consensus meetings with the coding team, with the last author, a Latino clinical psychology professor, available to adjudicate coding discrepancies. Coding discrepancies primarily involved clarifying definitions and identifying new categories to more accurately capture responses. Responses that were not consistent with the codebook definitions were also discussed during consensus meetings. When responses were too vague or unclear, those statements were categorized as “Other” (*n* = 20, 17%).

After double-coding all responses, each response was linked to the participant’s gender background, and the frequency of codes categories were then analyzed. Additionally, codes and categories were interpreted at the thematic level, which allowed us to contextualize our sample by understanding key challenges affecting MH help-seeking intentions, as well as gender differences among these challenges. Data saturation was reached after analyzing approximately 80% of the open-ended responses (*n* = 97), aligning with qualitative research guidelines indicating that saturation occurs when no new codes emerge and thematic redundancy becomes evident (Saunders et al., 2018).

## Results

### Sample Characteristics

The sample was roughly equal by gender, with women comprising 51.3% of the participants. The mean age of participants was *M* = 43.37 (*SD* = 19.01). Please refer to Table 1 for sample characteristics.

**Table 1:** Sociodemographic Characteristics

Variables		
	*N*	*%*
Gender		
Female	61	51.3%
Male	58	48.7%
Marital Status		
Single	47	39.5%
Married	53	44.5%
Divorced	6	5%
Separated	6	5%
Widowed	7	5.9%
Employment Status		
Part-time	12	10.1%
Full-Time	38	31.9%
Unemployed	35	29.4%
Not employed, looking for a job	13	10.9%
Not able to work	19	16.0%
Prefer not to answer	2	1.7%
Income		
$0-$999	16	13.4%
$10,000-$19,999	9	7.6%
$20,000-$29,999	19	16.0%
$30,000-$39,999	14	11.8%
$40,000-$49,999	13	10.9%
$50,000-$59,999	16	13.4%
$60,000-$69,999	9	7.6%
$70,000-$79,999	7	5.9%
$80,000-$89,999	4	3.4%
$90,000-$99,999	2	1.7%
$100,000 or more	8	6.7%
Prefer not to answer	2	1.7%
Previous Formal Mental Health Service Use (i.e., therapist, psychologist, psychiatrist, etc.)		
Yes	37	31.1%
No	80	67.2%
Prefer not to answer	2	1.7%
Previous Informal Mental Health Service Use (i.e., self-help group, spiritual advisor, herbalists, etc.)		
Yes	23	19.3%
No	94	79.0%
Prefer not to answer	2	1.7%
Hispanic, Latino, Spanish Origin		
Caribbean	2	1.7%
Central American	6	5.0%
South American	9	7.6%
Cuban	7	5.9%
Dominican	5	4.2%
Mexican/Mexican American/Chicano/a	65	54.6%
Puerto Rican	23	19.3%
Prefer not to answer	2	1.7%

### Aim 1: MHL and Stigma, with Gender Differences and Qualitative Contextualization

Please refer to Table 2 for a complete breakdown of the ANOVA analysis. Please refer to Table 4 for the complete qualitative code and frequency of codes.

**Table 2:** Means, standard deviations, and mixed-factorial ANOVA statistics for gender and time on MHL and stigma

Variables	Total (*n* = 119)		Female (*n* = 61)		Male (*n* = 58)		ANOVA			
	*M*	*SD*	*M*	*SD*	*M*	*SD*	Effect	*F* ratio	*df*	*n2*
Mental Health Literacy										
Pre-Intervention	6.60	2.00	6.49	2.01	6.71	1.91	T	10.438*	1,117	0.082
Post-Intervention	7.12	1.44	7.02	1.35	7.22	1.54	G	0.603	1,117	0.005
							G*T	0.001	1,117	0.000
Overall Stigma										
Pre-Intervention	21.50	8.09	20.84	7.12	22.21	9.01	T	3.774	1,117	0.031
Post-Intervention	22.49	8.51	23.13	7.91	21.81	9.11	G	0.000	1,117	0.000
							G*T	7.585*	1,117	0.061
Personal Stigma - Subscale										
Pre-Intervention	7.03	4.49	6.32	3.94	7.76	4.94	T	0.014	1,117	0.014
Post-Intervention	6.71	6.71	6.34	4.40	7.10	5.01	G	1.854	1,117	0.016
							G*T	1.890	1,117	0.016
Perceived Stigma - Subscale										
Pre-Intervention	14.48	5.62	14.51	5.34	14.44	5.95	T	0.089**	1,117	0.089
Post-Intervention	15.77	6.08	16.79	5.64	14.71	6.38	G	1.147	1,117	0.010
							G*T	7.252*	1,117	0.058

*N* 111, *ANOVA* Analysis of Variance, *G* Group, *T* Time, n2 effect size, *p < .05, **p < .01

#### Mental Health Literacy

We observed a significant main effect for time, *F*(1, 117) = 10.439, *p* = .002, partial η2 = 0.082, indicating that MHL scores significantly increased from pre- to post-intervention. There was no significant main effect for gender, nor did we observe a significant interaction between time and gender on MHL.

#### Regression and Qualitative Findings

We observed no gender differences in MHL changes following the presentation of the video. Table 3 Our qualitative results offer potential explanations for this finding. Specifically, the frequency of codes related to MHL—such as lack of knowledge about mental health challenges and limited awareness of treatment access and availability—was low, emerging in only four of our participants. Furthermore, the frequency of these codes was similar between Latinx female and male participants. See Table 4 for the complete frequency of codes.

**Table 3:** Hierarchical regression result for Help-Seeking intentions

Variable	B	95% CI for B		SE B	B	*R* ^2^	Δ *R*^2^
		LL	UL			0.652	0.009
Step 1							
Constant	3.987	1.292	6.679	1.359			
Baseline Help-Seeking Intention	0.709**	0.611	0.806	0.049	0.817		
Sex	0.375	− 0.561	1.311	0.472	0.045		
Marital Status	0.092	− 0.341	0.525	0.218	0.024		
Employment Status	− 0.151	− 0.522	0.221	0.187	− 0.046		
Income	0.077	− 0.080	0.234	0.079	0.055		
Step 2							
Constant	4.186	1.459	6.913	1.376			
Baseline Help-Seeking Intention	0.708**	0.610	0.805	0.049	0.816		
Sex	0.279	− 0.689	1.248	0.489	0.034		
Marital Status	0.052	− 0.381	0.485	0.219	0.014		
Employment Status	− 0.166	− 0.537	0.204	0.187	− 0.051		
Income	0.089	− 0.068	0.247	0.079	0.063		
Stigma	0.012	− 0.075	0.099	0.044	0.015		
Mental Health Literacy	0.217	− 0.033	0.466	0.126	0.097		

*CI* confidence interval, *LL* lower limit, *UL* upper limit, *R*^*2*^ effect size, ***Δ R***^*2*^ R-square change

p *< 0.001

**Table 4:** Summary of themes, categories, definitions, exemplar quotes, and response extensiveness by gender

Themes	Category	Definition	Exemplar Quotes	Sample Size	Male	Female	
Limited Mental Health Literacy is a Barrier to Seeking Mental Health Care	Knowledge about Mental Illness	Lack of knowledge about mental health symptoms and root causes.	“Getting to the core reason why someone may have a problem with mental health.” “Hard to find the root cause and there are no miracle cures.”	4	2	2	
	Knowledge about Mental Health System	Lack of knowledge about identifying and navigating available mental health services.	“People don’t know where to find it [services].” “Trying to figure out where or whom to go to.” “You don’t know… what to do to look for help.”	4	3	1	
Stigma is a Barrier to Seeking Mental Health Care among Latinx Adults	General	General discussion about stigma being associated with mental health.	“Seeking mental health treatment [is] difficult due to the stigma associated with it.” “There is a lot of stigma attached to mental health.”	6	3	3	
	Personal Stigma	Participants’ own negative beliefs or attitudes toward mental health and help-seeking, including internalized shame.	“Being ashamed of letting people know your inner issues that you’re dealing with because in your mind you feel you’re a freak” “Feel like most people feel embarrassed of their mental challenges and do not receive mental health care because of it.”	12	9	4	
	Perceived Stigma	Participants’ beliefs about how others (e.g., family, peers, or cultural groups) negatively view mental health and seeking support, including judging, ridiculing, or labeling.	“No one cares for people with mental illness, government officials will not get them off the streets and institutionalize them to get the help they desperately need to be productive citizens.” “I think that the person having the mental problems will have a hard time accepting that something might be wrong because mental illness is not talked about enough in the Hispanic community. Forget support. People are always on their own with this which might be hard for them to seek help because everyone will always say ‘get over it.’”	39		12	17
Structural Barriers Impact Seeking Mental Health Care among Latinx Adults	System-level Access Barriers	Barriers related to the accessibility of mental health care, including geographic limitations and systemic logistical issues.	“If only it was easier to get started, say by online, and by avoiding the long wait times and paperwork process, it could potentially be much easier to accept receiving help.” “services nearby and available”	10		5	5
	Lack of Providers	Insufficient availability of mental health professionals, including difficulties finding a good provider fit (e.g., relatable, trustworthy, or culturally responsive)	“Average mental health care professionals are frauds. [They’re] out to make money or virtue signal. They do not have proper training or compassion or intrinsic knowledge to help people.” “Finding a therapist you can relate to. Also many psychologists do not have openings.”	19		8	11
	Financial Barriers	Difficulties affording treatment, including lacking insurance or limited coverage for mental health services.	“The financial aspect or insurance that may cover some of the cost.” “With anything, the affordability. It’s hard to get affordable healthcare nowadays.”	25		14	11
Internal and Interpersonal Barriers Impacted Seeking Mental Health Care	Admitting Need for Help	Difficulty recognizing mental health issues and being ready to seek help, often involving delays in acknowledging the need for care and preparing for treatment.	“Accepting that you have a problem is the biggest challenge I have seen thus far. No one wants to admit something is wrong with them.” “Admitting that you have one [mental health problem] to begin with and that it is okay to seek help. I would never seek help, that would give me even more anxiety.”	27		12	15
	Psychological Barriers	Psychological symptoms and emotional responses that interfere with help-seeking behaviors, including depression, lack of motivation, and avoidance.	“When you are depressed you become stuck in a deep spiral of depression and you don’t want to change sometimes.” “The biggest challenge when accessing mental health care is anxiety and depression. Both can be severe and long lasting and have a big impact on one’s ability to get on with life.”	10		6	4
	Self-Disclosure	Difficulty sharing personal feelings or challenges, and expressing vulnerability.	“It’s very difficult to open up and reveal your inner self.” “Sometimes you can’t explain how you feel or even why you’re feeling that way.”	45		19	26
	Lack of Support	Absence of emotional, instrumental, or motivational backing from others when seeking mental health support.	“Walking inside a Mental Health facility, alone, to talk to someone about any problems I may be having.” “I believe it can be difficult if you are not on a supportive environment.”	18		9	9

#### Stigma

We did not find a significant main effect for time, nor for gender on pre-post total stigma scores. However, we observed a significant interaction between time and gender, *F*(1, 117) = 7.585, *p* = .007, partial η2 = 0.061. Simple effects tests revealed that stigma scores significantly increased from pre- (M = 20.84, SE = 0.91) to post-intervention (M = 23.13, SE = 1.01), *F*(1, 60) = 8.24, *p* < .01, among female participants, but not for male participants. Given the unexpected significant increase in stigma scores for female participants, we conducted a subscale-level analysis to determine whether personal vs. perceived stigma contributed to the increase in stigma scores. When testing the interaction on personal stigma, our analyses did not produce significant main effects for time and gender. However, when testing the interaction on perceived stigma, our analysis revealed a significant main effect for time, F(1, 117) = 11.441, *p* < .001, but not for gender. The analysis revealed a significant interaction between time and gender, F(1, 110) = 7.276, *p* = .008, η2 = 0.058 on perceived stigma. Simple effects indicated a statistically significant increase in perceived stigma scores from pre- (M = 14.51, SD = 5.34) to post-intervention (M = 16.79, SD = 5.64), F(1, 60) = 13.591, *p* < .001, among female participants, but not for male participants.

#### Qualitative Findings

Consistent with the quantitative finding, perceived stigma varied by gender with more female participants (*n* = 17) than male participants (*n* = 12) described how others judge, dismiss, or label individuals with MH conditions; perceptions that were often attributed to Latinx cultural norms that discourage emotional vulnerability, promote self-reliance, and associate mental illness with personal or familial weakness. For instance, a participant noted “we as Latinos are told from a young age to be strong and independent, to brush it off and keep going,” and another shared that, “depression is not talked about enough in the Hispanic community…[.]everyone […] say ‘get over it.

### Aim 2: Associations Between MHL, Stigma, and Help-Seeking Intentions, with Qualitative Insights Into Additional Influencing Factors Among Latinx Participants

Please refer to Table 3 for the complete results of the hierarchy regression.

#### Quantitative Findings

Our hierarchical multiple regression analysis revealed that changes in MHL (b = 0.012, *p* = .793) and stigma (b = 0.193, *p* = .153) did not significantly predict help-seeking intentions after the intervention.

While changes in MHL and stigma did not increase help-seeking, our qualitative findings revealed both influence help-seeking. Beginning with MHL, participants (*n* = 8; 7%) described how limited understanding of MH conditions and the healthcare system hindered their ability to access services. Some expressed confusion about identifying the “root cause” of MH challenges, noting that “most people don’t even know what’s going on with them,” while others struggled with knowing “where to find help.” One participant emphasized that navigating services becomes “easy if you have enough information to properly speak about mental illness,” highlighting how knowledge about MH conditions is closely tied to care access. These findings underscore the need for clear, accessible information about symptoms and system navigation to improve MHL and support help-seeking among Latinx individuals.

Similarly, our qualitative findings highlight how participants reported that stigma negatively impacts help-seeking behavior. First, participants (*n* = 6) described MH as “taboo”, emphasizing “there is a lot of stigma attached to MH,” reflecting general societal MH stigma. Participants (*n* = 12) then discussed personal stigma, describing internalized negative MH beliefs and help-seeking, with some expressing reluctance to “seem weak,” feeling “a lot of shame in asking for help,” and a desire “to be normal.” Lastly, participants (*n* = 29) described perceived stigma, explaining how others (i.e., family, peers, or broader cultural groups) view MH and professional help-seeking influence their willingness to seek care.

#### Structural Barriers

While our quantitative results did not show MHL and stigma scores predicting help-seeking, qualitative findings revealed additional barriers beyond MHL and stigma. Structural barriers emerged as salient obstacles (*n* = 54; 45%). First, participants (*n* = 10; 8%) highlighted system-level access barriers to MH care, such as a lack of service “close to where you live,” difficulty in securing “nearby and available appointments,” as well as “not having someone answer the phone.” Work conflicts hindered “going to appointments” or “trying to get in to see someone” challenging. Others emphasized the burden of navigating the system, noting that “if only it was easier to get started [.] by avoiding the long wait times and paperwork process.”

Participants then discussed limited provider availability (*n* = 19, 16%) created additional obstacles to accessing support. Namely, participants discussed difficulties locating providers who were culturally responsive, “relatable”, “trustworthy and helpful,” or aligned with their specific needs. Several participants expressed concerns about providers’ lack of empathy, compassion, and ability to build trust and rapport, while others expressed skepticism about providers’ “proper training.” Some participants described the search for getting the “right help” as discouraging and limited by a lack of viable options for effective care. Lastly, participants cited affordability (*n* = 25; 21%) as a barrier to care, noting both the high cost of treatment and lack of insurance. They described challenges such as “it’s hard to get affordable healthcare nowadays,” and noted that “not everybody can afford it.”

#### Internal & Interpersonal Barriers

Additional barriers identified through qualitative analysis included internal and interpersonal factors (*n* = 100; 84%) that hindered Latinx adults from seeking MH care. Psychological symptoms such as apathy, fear, shame, and low motivation were cited by 8% of participants (*n* = 10) as major obstacles. They emphasized the emotional difficulty of “having the courage to ask for help” and the struggle to prioritize MH when experiencing depression or anxiety. Internal resistance to acknowledging the need for help was noted by 23% of participants (*n* = 27) as another significant barrier. Many reported difficulties recognizing their MH challenges and reaching a point of readiness to seek care. One participant explained, “The biggest challenge… is for the person to come forward and finally accept that they may have a problem.” Others highlighted the emotional toll of this realization: “Admitting that you have one [a MH problem]… I would never seek help, that would give me even more anxiety,” and “Accepting that you have a problem is the biggest challenge I have seen thus far. No one wants to admit something is wrong with them.”

Lastly, 38% of participants (*n* = 45) identified difficulty expressing emotions or sharing vulnerabilities as a key barrier to seeking MH care—especially without supportive or understanding environments (*n* = 18; 15%). Many struggled with “opening up,” particularly to strangers, with one stating, “It’s hard to let a stranger into what you are experiencing.” Self-disclosure was especially challenging without emotional support or validation. Participants voiced frustration over not feeling heard, as one noted, “telling people over and over what’s going on… it’s frustrating,” and another shared, “not listening to what I’m trying to explain” led them to “shut down.” Several described the emotional toll of facing MH struggles alone—such as entering a “facility alone” or fearing being misunderstood. As one participant put it, “It’s hard to be like this because I have it myself [MH concern] and no one understands how I feel about it.”

## Discussion

We aimed to assess the effectiveness of a community-informed, culturally relevant video in increasing MHL and decreasing stigma among Latinx adults, and whether changes in both would lead to an increase in help-seeking intentions. Also, given the literature on gender differences in MHL and stigma, we sought to examine whether there were gender differences in MHL and stigma following the video intervention. Additionally, we conducted qualitative analyses to further understand and contextualize our quantitative findings.

As anticipated, the video-based psychoeducational intervention—developed specifically for the Latinx community—proved effective in enhancing MHL among Latinx adults across genders. This outcome aligns with existing research demonstrating the efficacy of video-based interventions in improving MHL (Lopez et al., 2009; Nazari et al., 2023). A likely explanation for this improvement lies in the intervention’s focus on key components of MHL., such as the ability to recognize MH symptoms, understand available treatment options, and appreciate the benefits of psychotherapy. For instance, the video portrayed common symptoms of depression (e.g., changes in appetite, low energy) and included scenes in which the main character initiates treatment and discusses its advantages. This design choice is supported by prior findings that interventions emphasizing symptom identification and treatment engagement increase MH service use among Latinx populations (Cabassa et al., 2012; Dueweke & Bridge, 2017).

Our qualitative data further emphasized the importance of these components. Participants identified limited MHL as a significant barrier to seeking care, with two primary subthemes emerging: a lack of knowledge regarding MH symptoms and insufficient awareness of how to access MH services. While the intervention addressed the concept of psychotherapy and its benefits, it did not provide a detailed guide for navigating the process of accessing care. This gap was reflected in participants’ feedback, as many expressed a desire for more concrete information about how to seek services. These findings align with previous research that calls for more practical, user-friendly psychoeducational materials to support navigation of MH systems (Aguilar Silvan et al., 2024).

Contrary to prior research suggesting that video-based psychoeducational interventions are effective in reducing stigma toward mental illness (Corrigan et al., 2011; Wong et al., 2019), our study did not observe a significant overall decrease in stigma following the intervention, nor were there notable gender differences in stigma reduction. Interestingly, however, we found a significant increase in perceived stigma among Latinx female participants. One potential explanation is that the video may have inadvertently reinforced awareness of stigmatizing beliefs held by others. For instance, a scene in which the protagonist, Victor Luna, is dismissed as “lazy” by his parents, may have heightened participants’ sensitivity to societal stigma. Without sufficient narrative framing, such depictions risk amplifying perceptions of public stigma. This finding is consistent with literature suggesting that women are generally more attuned to social cues and more likely to perceive and report stigma (Busby et al., 2016).

Supporting this, our qualitative analysis revealed that more women than men identified perceived stigma as a barrier to care, which may further reflect heightened sensitivity to social dynamics and stigma-related messaging. Additionally, while the intervention was not explicitly designed with a male audience in mind, the use of a male protagonist may have inadvertently fostered greater identification and relatability among male participants, potentially buffering them against increases in perceived stigma. Although the video was intended to portray the lived experience of a Latinx male authentically, our findings suggest that a testimonial-style approach—particularly one incorporating direct contact or lived-experience narratives—may be more effective in reducing stigma. Prior research supports the effectiveness of contact-based interventions, especially those involving live or interpersonal engagement, as these allow for real-time modeling of non-stigmatizing attitudes and opportunities to correct misconceptions (Thornicroft et al., 2016). These findings highlight the need to tailor stigma-reduction efforts not only to cultural context but also to gender-specific experiences and interpretations of MH content.

Lastly, we did not observe significant changes in help-seeking intentions as a result of changes in mental MHL or stigma. Our findings suggest that these changes in MHL and stigma alone may not be sufficient to influence help-seeking behavior within the Latinx community. The absence of significant effects underscores the need to examine additional, potentially more influential, barriers and facilitators of help-seeking. Our qualitative data provide critical insight into these dynamics. Participants reported a variety of structural barriers, including limited availability of MH services, a lack of culturally responsive providers, and financial constraints. They also identified internal and interpersonal obstacles such as difficulty acknowledging the need for help, discomfort with emotional disclosure, lack of social support, and psychological resistance to treatment. These themes align with existing literature documenting similar barriers to MH service utilization (Benuto et al., 2020; Cho et al., 2014; Gearing et al., 2023). Moreover, recent findings by Saravia et al. (2025) suggest that stigma may be less central to Latinx individuals’ decisions to seek care than previously assumed (ranked lowest relative to other factors impacting service use). These insights highlight the complexity of help-seeking behavior and reinforce the necessity of addressing a broader range of cultural, structural, and psychological barriers in future interventions.

## Limitations & Strengths

The non-significant association between changes in MHL and stigma with help-seeking intentions may be partly due to the lack of temporal precedence, as data were collected only ~ 20 min post-intervention. Prior studies suggest attitudinal shifts may emerge more clearly over time (e.g., four-week follow-up; Thornicroft et al., 2016). Measurement limitations may also have played a role. While the stigma and help-seeking intention measures demonstrated strong internal consistency, the MHL measure showed lower reliability. This lower internal consistency should be interpreted in context. First, the MHL measure was adapted for clarity by omitting 11 items; reducing the number of items can attenuate reliability estimates, as internal consistency is partially dependent on scale length. Second, the MHL measure consists of dichotomous (true/false) items and functions more as a checklist of discrete knowledge components rather than a unidimensional scale. In such measures, participants may correctly endorse some items but not others, resulting in lower inter-item correlations and, consequently, lower reliability estimates (e.g., KR-20).

Additionally, both MHL and stigma measures focused solely on depression, while the video focused on internalizing symptoms more broadly. Future studies should use broader measures to better capture intervention effects. Generalizability may be limited due to the sample’s specific characteristics: English-speaking Latinx adults with computer access. Further, we did not assess participants’ current MH needs, which may have influenced help-seeking intentions.

Despite its limitations, this study possesses several notable strengths. First, participant recruitment was conducted with intentionality and focus. Recruitment efforts were deliberately limited to Latinx individuals without a college degree, a population that represents a significant proportion of the broader Latinx community— (Office of Minority Health, 2024). This focus is critical, as research has consistently shown that adults with lower levels of educational attainment are less likely to access MH services (Halme et al., 2023). Prioritizing the inclusion of this underserved group enhances the relevance and applicability of the study’s findings. Methodologically, the use of a mixed methods design represents an additional strength. This approach enabled us to examine not only the impact of the intervention video but also the mechanisms through which changes in MHL and stigma may influence help-seeking intentions—an area that remains underexplored in existing literature. Furthermore, the integration of qualitative data provided deeper insight into unexpected outcomes and helped identify additional barriers to help-seeking within the Latinx community.

### Clinical Implications and Future Directions

Video-based psychoeducational interventions offer a scalable and accessible method to enhance MHL and reduce stigma in Latinx communities. This study demonstrated that such interventions can effectively improve MHL by presenting symptoms of MH conditions, available treatments, and the benefits of care. However, participants expressed a need for more practical guidance on navigating the MH system, suggesting that future interventions should include information on how to access services. Despite improvements in MHL, the intervention did not reduce stigma and, unexpectedly, increased perceived stigma among female participants. This highlights the importance of explicitly addressing and correcting common misconceptions. Incorporating targeted segments that explain clinical symptoms may help challenge these beliefs and reduce stigma, ultimately supporting greater openness to care. While the intervention aimed to increase help-seeking intentions by improving MHL and reducing stigma, this was not observed. Qualitative findings and prior literature suggest that other factors—such as perceived need for treatment, family influence, and systemic barriers—may play a more critical role in help-seeking behaviors among Latinx individuals. Future interventions should address these factors and include culturally relevant strategies, such as promoting family involvement and highlighting low-cost or free MH resources. Furthermore, the intervention focused solely on formal help-seeking and did not assess intentions to seek informal support from family, peers, or community-based networks—an important consideration given the Latinx community’s reliance on informal sources of support. Future research should examine how psychoeducational materials influence both formal and informal help-seeking to better align with culturally grounded patterns of care. Although the video was developed using community-partnered, participatory research principles with input from MH providers, deeper engagement with target audience members during development could have strengthened the intervention’s cultural and contextual relevance. Collecting qualitative feedback on aspects such as character relatability, video length, and production quality could inform refinements to improve engagement and effectiveness.

## Conclusion

This study contributes to the growing body of research on video-based interventions aimed at increasing MHL and reducing stigma, and in turn increases help-seeking within Latinx communities. Advancing MH equity requires culturally responsive strategies, and our findings offer a foundation for developing effective, community-informed video content. Integrating core MHL principles—such as symptom recognition, treatment awareness, and service navigation—can significantly enhance MHL by providing accurate and accessible information. While narrative formats increase relatability, they must also explicitly confront stigma to avoid reinforcing harmful beliefs. This study serves both as a proof of concept and a blueprint for future researchers and intervention developers, highlighting what elements are effective and which require refinement.

## Data Availability

No datasets were generated or analysed during the current study.
